# Reassessing data quality underlying the recently updated floristic map of the world

**DOI:** 10.1038/s41467-024-47543-7

**Published:** 2024-05-02

**Authors:** Hong Qian

**Affiliations:** Research and Collections Center, Illinois State Museum, 1011 East Ash Street, Springfield, IL 62703 USA

**Keywords:** Biodiversity, Plant ecology

**arising from** Y. Liu et al. *Nature Communications* 10.1038/s41467-023-38375-y (2023)

Generating maps of floristic and faunistic regions based on native distributions of organisms is an important task in biogeography^1,2^. Recently, ref. ^3^ generated a set of global maps of floristic realms. When reassessing the data used in their study, I found that most of the geographic units included a large number of non-native genera, while many native genera were not included. These problems could invalidate the maps and analyses reported in the study.

Alfred Russel Wallace, Augustin Pyramus de Candolle, Chevalier de Lamarck, Joachim Frederik Schouw, and Adolf Engler were among the pioneers who generated biogeographic maps^1^. Earlier work on biogeographic regionalization focused on the taxonomic ranks of genus and family. Recently, information on evolutionary (phylogenetic) relatedness among different taxa has been used when generating biogeographic maps (for example, ref. ^2^). Regardless of which approach (taxon-based or phylogeny-based) is used in generating a biogeographic map, the quality of data for the native distributions of each taxon directly affects the quality of the resulting biogeographic map.

Liu et al.^3^ integrated distribution data and phylogeny of 12,664 angiosperm genera distributed in 420 geographic units to generate a global map of floristic realms. They explored the mechanisms that have driven the formation of the identified floristic realms by evaluating the effects of contemporary climate, the dynamics of geographic isolation and historical climate, as well as the relative contributions of lineage-splitting events at different geological times to realm divisions. The quality of the floristic map they have generated and the robustness of the conclusions of their study depend heavily on the quality of the data used in their study. I found substantial flaws in the data used in ref. ^3^, which suggest they are not appropriate for generating a floristic map to reflect native distributions of plants. Below I highlight two of the major problems with their study.

Many distribution occurrences of genera in the geographic units used in ref. ^3^ are not native. Of the 12,664 genera in their study, 11,747 (93%) are accepted genus names in the World Checklist of Vascular Plants (WCVP), which can be accessed via Plants of the World Online (POWO) at https://powo.science.kew.org/^4^. For each genus, POWO provides a global map showing distributions in geographic units in up to four statuses (native, introduced, extinct and doubtful). I compared distributional data reported by ref. ^3^ with those in POWO and found that ref. ^3^ included non-native (introduced) genera in each of the 420 geographic units used in their study. Over 200 geographic units included more than 100 non-native genera in each unit. For example, over 700 genera that ref. ^3^ considered as native to Myanmar are actually not native to Myanmar, according to POWO. Of the 11,747 genera accepted both by ref. ^3^ and by POWO, 7562 have at least one non-native distribution occurrence in the distribution data used by ref. ^3^. In some genera, non-native distributions were mistakenly considered as native distributions in over 100 geographic units. In the case of *Zea* (commonly known as maize and corn), 99% of the distribution occurrences used in ref. ^3^ are non-native distributions, and their occurrences in these geographic units reflect cultivation (Fig. 1f, l). Another example is the genus *Tamarindus*. According to POWO, this genus is endemic to Madagascar (Fig. 1k), but ref. ^3^ considered it as native in over 120 geographic units located in mainland Africa, Asia, Australia, North America and South America (Fig. 1e). The aforementioned genera are only two of the over 7500 genera that have a mixture of native and non-native distributions in ref. ^3^. These non-native genera compose of over 63,000 non-native occurrences in the data used in the study, all of which were identified based on the POWO plant database. Liu et al.^3^ state that they checked the distribution maps and removed cultivated records from their database following POWO, but the fact that there are so many discrepancies in genus origin status (native versus non-native for a given genus in a particular geographic unit) between the data used in the study and the POWO database casts doubt on the study by ref. ^3^.

**Fig. 1:**
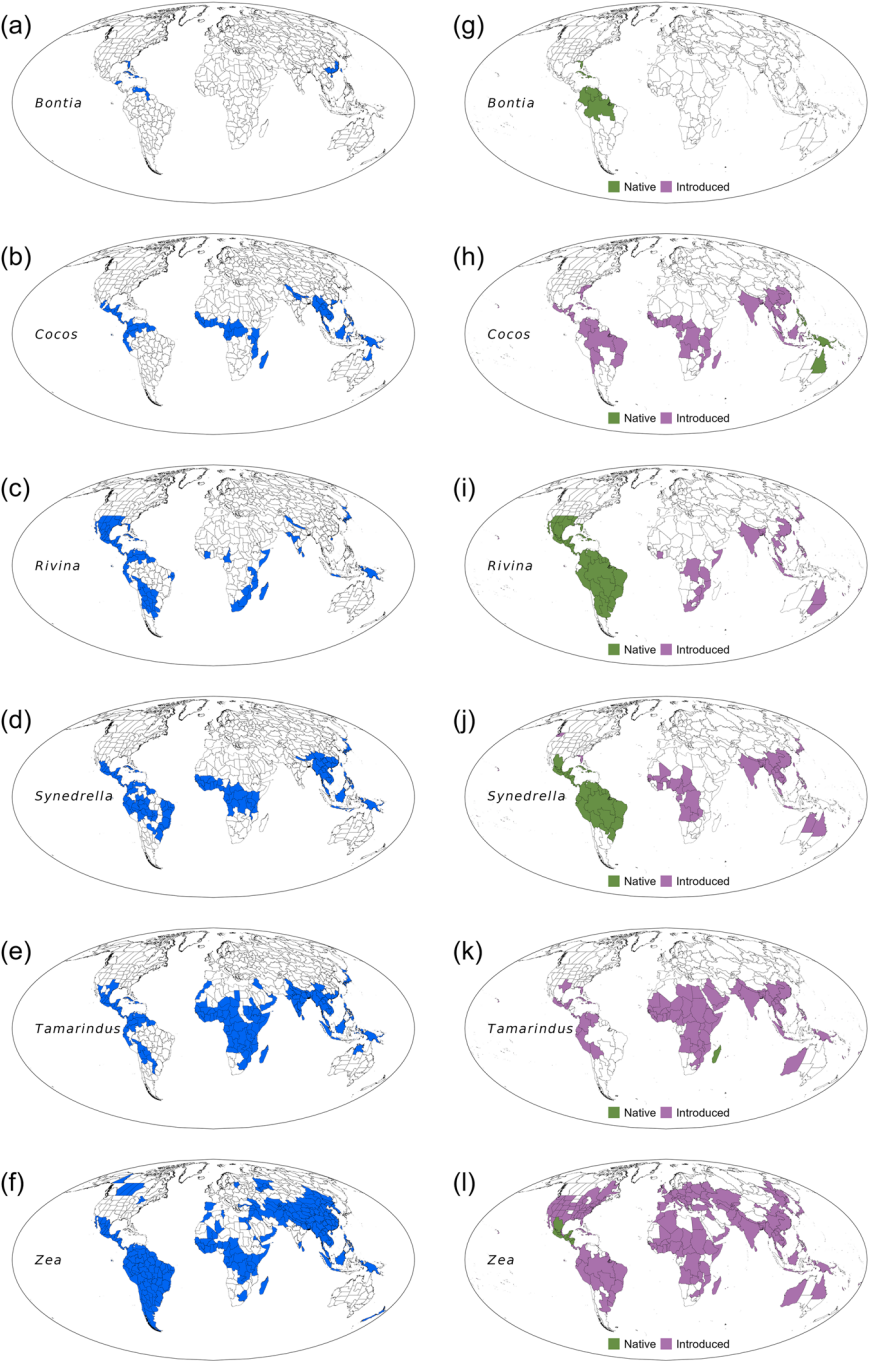
Global distributions of selected genera of plants. Six examples of genera used in ref. ^3^ that included abundant non-native (introduced) distributions. In each of the six maps on the left column (**a**–**f**), regions in blue indicate distributional data used in ref. ^3^, which are a mixture of native and non-native distributions, as shown in the distributional data in the six maps on the right column (**g**–**l**), which were derived from Plants of the World Online (POWO; https://powo.science.kew.org; assessed via the package rWCVP by ref. ^4^).

Checklists of angiosperm genera are substantially incomplete for many of the geographic units used in ref. ^3^. Of the 420 geographic units used, 132 can be perfectly matched with those in POWO. None of these geographic units have complete genus lists in ref. ^3^ and incompleteness is >40% in some of the geographic units. For example, Maluku Islands had 518 genera (including 93 introduced genera based on the information at POWO) in ref. ^3^ but there are 1014 genera of native angiosperms in POWO. Other geographic units in ref. ^3^ that had <60% completeness of their genus lists include Sulawesi Island, Tasmania, Java Island and Lesser Sunda Islands (geographic unit names are adopted from ref. ^3^).

Using regional genus lists that include abundant non-native genera on the one hand, but are substantially incomplete on the other hand, would likely have substantially biased the results and conclusions of ref. ^3^. In particular, because the conclusions heavily depend on phylogenetic relatedness among genera, including only few non-native genera in a geographic unit may cause severe problems and substantial biases in the results. This is particularly the case if a non-native genus belongs to an evolutionarily distinct and ancient lineage introduced from a different continent, because genera and families evolved in different biogeographic regions often have quite different evolutionary histories. When an ancient lineage (for example, a family or order) that is endemic to one continent is introduced to another continent, and if both native and non-native distribution occurrences are included in a phylogeny-based study that requires only native distributions, including few such lineages can cause a bias because native and non-native lineages in a continent might diverge at a deep node of the phylogeny. Such problems indeed occur in ref. ^3^. For example, *Eucommia* is a monotypic genus belonging to the family Eucommiaceae, which is in turn a monotypic family (i.e., only one genus in the family). The family Eucommiaceae diverged from its sister family Garryaceae ca. 76 million years ago^5^. Thus, *Eucommia* is an evolutionarily distinct and ancient lineage, and native only to eastern Asia. However, ref. ^3^ included non-native distributions of *Eucommia* in North America (Costa Rica and Panama, Indiana, Kentucky, and Ohio) in their study. Similar problems occur in many other families. For example, *Cercidiphyllum* is the sole genus of the family Cercidiphyllaceae, which is an ancient lineage^6^ and endemic to eastern Asia, but Liu et al.^3^ included non-native distributions of this family in North America in their study. Other ancient families that are endentic to Asia but were mistakenly treated as native in other continents in ref. ^3^ included Trochodendraceae and Daphniphyllaceae^5^.

In summary, generating a high-quality floristic map requires good-quality native plant distributional data. Genus lists for the geographic units used in ref. ^3^ are a mixture of native and non-native genera, and some geographic units each included hundreds of non-native genera. I found that most (~60%) of the genera used in their study included non-native distributions (over 63,000 non-native occurrences). This problem alone is sufficient to invalidate the floristic maps and any related analyses, not to mention that the composition of angiosperm native genera documented by ref. ^3^ is also incomplete for most of the geographic units used in their study.

## Methods

Distributional data of ref. ^3^ were obtained from Supplementary Data 2 of their article. Distributional data of Plants of the World Online (POWO) were obtained from the website https://powo.science.kew.org, using the package rWCVP by ref. ^4^. To determine the origin status of each genus in each geographic unit in the data from ref. ^3^, I matched the geographic units of the study with those of POWO. In cases in which a geographic unit of ref. ^3^ comprises two or more geographic units of POWO, if a species is considered as native in any of the geographic units of POWO, I considered the species as native in the geographic unit of ref. ^3^. Similarly, if a geographic unit of POWO is composed of two or more geographic units of ref. ^3^, and if a species is considered as introduced in the geographic unit of POWO, I considered the species as non-native in all the geographic units of ref. ^3^. For a geographic unit matched between ref. ^3^ and POWO, if a genus is present in ref. ^3^ but absent from POWO, I considered it as non-native to the geographic unit, assuming that native distribution data in POWO are, in general, up-to-date.

### Reporting summary

Further information on research design is available in the Nature Portfolio Reporting Summary linked to this article.

### Supplementary information

Reporting Summary

## Data Availability

No datasets were generated for this manuscript. Distributional data of ref. ^3^ were obtained from Supplementary Data 2 of their article. Distributional data of Plants of the World Online (POWO) were obtained from the website at https://powo.science.kew.org.
